# Perineorraphy

**Published:** 1878-09

**Authors:** 


Perineorraphy.—I regard the operation for restoration of the female perineum as a very important one, for the reason that the opportunities for performing it are so numerous.

There is no operation that the gynæcologist is required to perform so frequently; and if he succeeds in doing what he attempts (restoring the perineum), the value of his services to the unfortunate female cannot be over-estimated.

My apology for reading a paper before so learned a body, on so common an operation, is, that on examination, I have met with so many cases operated on by myself and far more skillful surgeons, in which the condition of the patient was but little, if any, better than when she first applied to the surgeon for treatment. And it is because I have reason to be better satisfied with my results for the last few years, that I trespass upon your time to explain the way in which I do the operation now.

It would be out of place for me to take up your time with the causes of rupture of the perineum, but I wish just here to say that most of the cases that I have seen were the result of the use of the forceps, and most of them occurred at the first confinement; so that for years it has been my invariable rule to remove the instrument as soon as the child's head distends the perineum, in a first labor. By so doing a little time is lost, but the perineum is often saved.

In my judgment, far too little attention is paid to the treatment of the patient previous to the operation. In many of the cases the womb is in a condition of complete prolapse, and has been so for a long time. All the supports of the uterus are in an abnormal condition; the ligaments are stretched, the vagina is everted, the womb, from congestion, is too heavy. Now if, as is often the case, the operator returns the organ within the pelvis and closes the perineum, it will be but a very short time before the uterus will stretch the newly-formed tissue apart, and find its way outside the body again; but if, by the use of appropriate remedies, the uterus be relieved of the congestion and resulting hypertrophy, and if, by the use of a pessary supported externally, the organ be kept in position till the vagina recovers its tone and the ligaments are in a condition to help hold the uterus up, the results of the operation will be very different; and if months are required to accomplish this, the time is not lost. It is my practice to continue to hold the womb up off the parts for some time after restoring the perineum, in order that the union may attain firmness sufficient to resist pressure from above, should there be any.

I think there is no operation the reports of which are so well calculated to mislead the young practitioner as this one. All, or nearly all, are reported as perfect successes, and honestly so, for the report is made just after the operation, when the external appearance of the parts is very much improved; the long, gaping opening is replaced by a well-closed vulva. Convinced of his success, the operator so reports it, when, if he had waited for a year, and then examined his patient, he would have found, in very many cases, that he had not restored the perineum at all, but had only united the skin for an inch or two, from which she had received little or no benefit, and had found herself, after a few months, as bad off as before the operation.

The union of the skin high up is a matter of very little importance compared with diminishing the capacity of the lower two inches of the vagina by a firm union, of a triangular shape; and, I believe, in a great majority of cases, that may be done, the patient having had the advantage of skillful treatment to put her in a proper condition, by operating in the following manner:

Let her be placed on her back upon a table, before a window, and when etherized, her legs are to be held in position by two assistants, who also, with the fingers of the free hand, stretch open the vulva. The operator sits before his patient, and, having removed the hair from the labia, introduces two fingers into the rectum, to put the parts upon the stretch, and, with the scissors curved on flat, he removes a thin layer of the mucous membrane, for a distance of two inches up the posterior wall of the vagina; then, dissecting from this line on either side, he denudes a triangular surface, which extends from the lower anterior edge for an inch and a half up the labia, and for two inches up the posterior wall of the vagina. Every part of this surface must be denuded, and it is important it should be done evenly, and the thinner the lamina taken off the better.

The hemorrhage is always slight, and it is better not to use ice or to throw a stream of water upon the parts with a syringe, nor is it well to close the wound and depend upon the sutures to stop the bleeding. I am in the habit of waiting half an hour, till all oozing stops, and the denuded surfaces present a glazy appearance, before introducing the sutures, and it is upon the way in which *this* is done that everything depends.

Iron wire, plated with silver, is less likely to break than silver wire, and is preferable, for that reason.

The first suture is a very important one, as you depend upon it to control the action of the muscles: it is introduced by entering the point of the perineal needle an inch from, and half an inch below, the lower angle of the wound, passing it across the middle of the vivified surface, and out at a corresponding point on the opposite side. This suture passes over the rectum and under the denuded surface, and is not seen in the vagina. The eye of the needle, which is at the point, is now threaded, and as the needle is withdrawn the suture is introduced. The second suture enters from half to three-fourths of an inch above, and is passed in the same way. All the other sutures are seen in the vagina, just at the edge of the vivified surface.

Now, if you pass both ends of the suture through a perforated shot, run it down and clamp it, you will be very much pleased with the appearance of the wound after it is closed in this way—but what will you do? You will pucker the whole mass up, instead of bringing the surfaces evenly together. You will be obliged to draw the wire so tightly that you will interfere with the circulation by this process of strangulation; and once the shot is clamped in this way, it cannot be removed; and as the part swells, as of course it will, if the wire does not cut through the skin (as it often does), it is because the mucous membrane within the vagina offers less resistance; and as there can be no union outside the sutures, all this is lost.

To obviate these difficulties, you are directed by some operators to clamp a shot on each end of the suture; but when I did this I found that the shot were pulled through the skin and the edges of the wound separated. So I made some little disks of lead, about the size of a silver three cent piece, punched a hole in them and ran them down, then ran the shot down and clamped it. This I found to answer the purpose perfectly, and I do away with the shot now by using disks of lead, made for me by Mr. Gemrig, with a nipple on them to be clamped, to hold them in place.

In this way you close the posterior edges of the wound, and the anterior edges are now brought together by taking the end of a suture in each hand, crossing them and twisting them twice around; in this way the parts are brought perfectly together.

I do not cut off the ends of the wires, but leaving them about three inches long, gather them in a bundle and wrap them with a piece of muslin, and as you find the swelling to increase, untwist the wires a little and give the parts more room, and after a few days, as the swelling decreases, twist them tighter from day to day, if they require it. In this way the parts are kept evenly together, and as the surgeon watches his bandages and prevents strangulation by giving more room when more is required, so you from time to time adjust your dressings, in accordance with the condition of the parts.

The bowels are kept closed with opium; the water drawn; the knees tied together. The sutures may be removed the seventh or eighth day. The bowels may be moved with care on the ninth or tenth day, and I think well of locking them up again for a week afterward. Of course all I have said here relates to ruptured perineum when the parts have cicatrized; in recent cases I am in favor of immediate operation, and I put the stitches in the same way.

My results since I have operated in this way have been very satisfactory, but they have not all been perfect successes, and from the many cases that I have met with at the clinic, and in my private practice, that have been operated upon by others, I am sure that I make no mistake when I say again, that those operators who never fail to restore the perineum would make a different report if they waited for a year, and then examined their patients.—*Medical and Surgical Reporter.*

